# Distinct intraspecific trait variations in two moss species: Insights from a latitudinal investigation across 66 coastal islands

**DOI:** 10.1016/j.pld.2025.12.016

**Published:** 2026-01-02

**Authors:** Zhe Wang, Sheng-Xuan Cai, Jing-Rou Yu, Dan-Dan Li, Xue-Ping Lai, Ling-Ao Yang, Shui-Liang Guo, Jing Yu

**Affiliations:** a Shanghai Normal University, Shanghai 200234, China; b Yangtze River Delta Urban Wetland Ecosystem National Field Scientific Observation and Research Station, Shanghai 200234, China

**Keywords:** Biogeography, Bryophyte, Functional trait, Morphology, Substrate, Water relations

## Abstract

As a non-negligible component of functional trait diversity, intraspecific trait variation (ITV) represents a direct response of local populations to environmental change and significantly influences ecological processes such as species dispersal, community assembly, and ecosystem functioning. Although extensively studied in vascular plants, ITV remains underexplored in mosses, which possess fundamentally distinct evolutionary status, structures, and adaptive strategies. In this study, we sampled two common subtropical terricolous and saxicolous moss species (*Hyophila propagulifera* and *Pogonatum inflexum*) from 66 coastal islands (spanning a latitudinal range of 23.4–30.9°N) in southeastern China, measured their morphological and anatomical traits and water holding and retention capacities, identified the effects of latitude and environmental factors on each trait, and calculated the relative contributions. For both species, the trait with the highest variation was shoot mass, whereas the shapes of the stem transverse section, leaf, and leaf cells were relatively conserved. In *H*. *propagulifera*, stem size and internal transport capacity increased along a latitudinal gradient, however, the water holding and retention capacities were not associated with any of the studied environmental factors. In *P*. *inflexum*, lamella length was negatively correlated with precipitation, whereas no clear ITV patterns were observed in other traits, probably because of Polytrichaceae’s unique characteristics and the more stable microclimates of soil substrates compared with rock. This work expands our understanding of the adaptive evolutionary strategies of poikilohydric plants and bridges knowledge gaps in their conservation from the perspective of functional adaptation. We suggest that future studies focus on the impacts of climatic events on mosses and combine functional traits with community structure surveys to accurately quantify their ecosystem functioning and services.

## Highlights

• Intraspecific trait variations are understudied in poikilohydric, nonvascular mosses.
• Two common terricolous and saxicolous mosses were sampled from 66 coastal islands.
• Intraspecific variation was determined for various traits, including water relations, morphological, and anatomical traits.
• Shoot mass varied the most, whereas shapes were relatively conserved.
• These two moss species showed distinct intraspecific trait variations along a latitudinal gradient.

## 1 Introduction

Plant functional traits indicate their growth performance and adaptations to the environment, and potentially reflect the roles these plants play in ecosystem processes and functioning (Violle et al., 2007). Trait diversity, both in form and function, is the focus of functional biogeography, which analyzes the patterns and causes of plant geographic distribution (Violle et al., 2014). Within this, intraspecific trait variation (ITV) is the most direct response of local populations to changing and heterogeneous environments, which is quantitatively non-negligible and shapes ecological processes including species dispersal and distribution, community assembly, and species coexistence, and ecosystem functions such as productivity and nutrient cycling (Albert et al., 2011). Although ITV has been widely studied in vascular plants, such variations remain critically underexplored in mosses (Bolnick et al., 2011; Des Roches et al., 2018). A recent global synthesis revealed that ITV is largely overlooked in moss trait ecology, with research effort being strongly biased geographically toward temperate and boreal regions and taxonomically toward peat mosses (*Sphagnum* spp.) (Coe et al., 2024). With a dominant gametophyte generation, mosses are non-vascular and poikilohydric plants. Lacking meristematic tissues, lignin, tracheids, and sieve cells, and with leaves often only one cell layer thick, their water status is largely controlled by environmental water availability (Glime, 2017). Due to the distinct differences in phylogenetic status, structures, and ecophysiological characteristics between mosses and vascular plants, ITV patterns found and validated in vascular plants may not be entirely suitable for mosses.

With relatively simple structures and a poikilohydric strategy, mosses are widely distributed across all continents, abundant in many harsh environments, and serve as effective and cost-efficient bioindicators (Geffert et al., 2013; Li et al., 2024; Han et al., 2025; Wang et al., 2025a). Exploring the biogeography of mosses contributes to predicting the impact of global climate change on them and provides insights for the conservation and restoration of their diversity and ecosystem functioning (Li and Prentice, 2024). However, the existing knowledge is mainly concerned with species spatial distribution, diversity, and abundance (e.g., Qian et al., 2024; Wang et al., 2026), while studies focused on variations in their unique functional traits, such as the morphology and anatomy of the one-cell-layered leaf and the poikilohydric plants’ water relations, remain limited and the patterns described are mostly across species (Stanton and Coe, 2021; Fu et al., 2024; Liu et al., 2024; Yang et al., 2025). Although interspecific trait variations efficiently capture broad adaptive trade-offs at the community level, conclusions drawn solely from these often mask the role of ITV in mediating local adaptation, population resilience, and community assembly (Shipley et al., 2016). Biogeographical studies on ITV in mosses have almost exclusively been conducted on arid species from inland plateau and desert regions (Wang et al., 2019a, 2024; Liu et al., 2021; Zhuo et al., 2023; Gu et al., 2024), but have rarely been extended to warmer and more humid regions, which harbor different species that experience distinct environmental stresses.

With clearly defined boundaries, quantifiable isolation (distance), area, and shape index, as well as varied geological histories and high habitat heterogeneity, islands are ideal for elucidating the mechanisms that underlie plant diversity, including biogeographical processes such as migration, diversification, and extinction (Whittaker et al., 2017; Schrader et al., 2021). Mosses are spore-producing plants that exhibit a strong capacity for long-distance dispersal, generally resulting in low island endemism, and their diversity patterns are less influenced by historical geographic isolation but more strongly driven by environmental factors (Patiño and Vanderpoorten, 2021). Nevertheless, this makes those widely distributed moss species on islands an excellent candidate to study ITV along environmental gradients. Such studies offer a functional perspective to reveal the ecological strategies of moss, thereby helping to understand how environmental filters govern species assembly and ultimately drive plant diversity in insular environments (Schrader et al., 2021).

To identify the factors (i.e., environmental and latitudinal) that drive ITV in mosses, we sampled and determined the morphological and anatomical traits and water relations of two common subtropical terricolous and saxicolous moss species across 66 coastal islands in southeastern China (from the Nanao Islands in the south to the Zhoushan Archipelago in the north), spanning a latitudinal range of 23.4–30.9°N and presenting an ideal environmental gradient for biogeographical investigation. By revealing the contrasting latitudinal ITV patterns between these two species, quantifying the relative contributions of the driving factors, and examining their specific effects on functional traits, this work expands our understanding of the adaptive evolutionary strategies of poikilohydric plants and addresses key knowledge gaps in their conservation from a functional adaptation perspective.

## 2 Methods and materials

### 2.1 Focal species, sampling locations, and environmental factors

From 2016 to 2019, the same field team conducted extensive bryophyte specimen collection and diversity surveys across 233 southeastern coastal islands in China. These islands span a latitudinal distance of approximately eight degrees, with areas ranging from 0.0028 to 502 km^2^. We employed a method similar to floristic habitat sampling, rather than plot-based sampling, to compile relatively comprehensive bryophyte species inventories and to ensure the comparability of species richness data (Li et al., 2023). Briefly, bryophyte specimens were collected across the entire area of small islands (< 0.01 km^2^). For larger islands (> 0.01 km^2^), we surveyed two roughly perpendicular transects across the center of each island, aiming to cover a wide range of landscape types. Moreover, we continued sampling along other accessible pedestrian routes, such as roads, streams, and fire trails, until no new habitat types or additional bryophyte species were recorded.

**Fig. 1:**
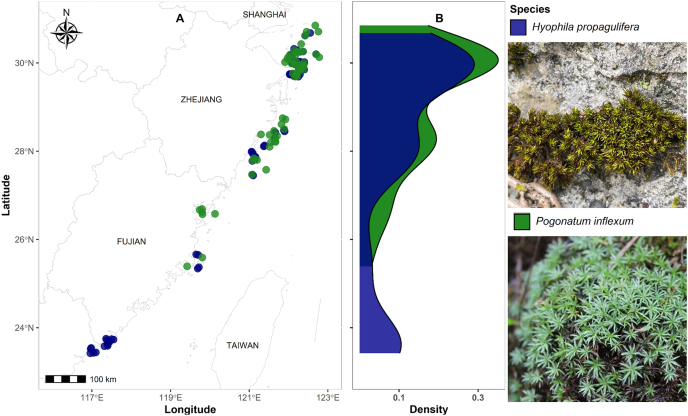
Mosses sampled on islands along the southeastern coast of China.

Based on the frequency of occurrence across islands and the abundance of collected specimens, we selected the most frequently encountered species. Notably, different growing substrates (e.g., rock and soil) may host distinct moss species, as these substrates provide different nutrient sources and quantities, as well as microclimatic conditions (Wang et al., 2019a, 2019b). Accordingly, two moss species were selected (Fig. 1): the small (*ca*. 5 mm in height), saxicolous *Hyophila propagulifera* Broth (Gao, 1996), which mainly grows on exposed, sunny rocks or concrete surfaces near buildings, and the medium-sized (*ca*. 2 cm), terricolous *Pogonatum inflexum* (Lindb.) Sande Lac. (Wu et al., 2005), which is mostly found on soils (red soils with a pH ∼5.2) at forest edges or roadsides. Both species have a turf life form and are widely distributed in the subtropical and warm temperate regions of China (Glime, 2017).

Based on the collection coordinates, we selected and used the specimens collected during field surveys as our study samples. For islands spanning less than 0.03° latitude, we defined one “sampling location” at the center of the island. The three specimens closest to this sampling location were combined as one sample representing this location. For islands with larger areas and longer latitudinal ranges, we set multiple sampling locations distributed evenly along the north–south gradient, with the number of locations proportional to the latitudinal range. For example, we set four sampling locations and collected *Pogonatum* *inflexum* samples from Zhoushan Island (Fig. S1). In total, we had 79 *Hyophila* *propagulifera* (from 42 islands) and 56 *P*. *inflexum* samples (from 49 islands) (Fig. 1 and Table S1). Mean annual temperature (MAT), mean diurnal range (MDR), and mean annual precipitation (MAP) of these sampling locations were extracted from the WorldClim website (https://www.worldclim.org). Additionally, ultraviolet B radiation (UVB) data were extracted from the global UV-B radiation dataset (Beckmann et al., 2014).

### 2.2 Measurements of water relations and shoot mass

About 20 medium-sized, intact shoots were selected from each sample and gently washed with distilled water to remove surface dust. The shoots were then soaked in water for 1 h to ensure complete saturation. Afterward, we placed the aboveground shoots in the laboratory to air-dry under stable conditions (approximately 25 °C and 60% relative humidity). Their fresh mass was measured and recorded at 6-min intervals until constant weights were obtained (about 100 min for *Hyophila* *propagulifera* to dry out and 75 min for *Pogonatum* *inflexum*) (Wang and Bader, 2018). The samples were then oven-dried at 70 °C for 24 h to obtain their dry mass and calculate the average mass of individual shoot (shoot mass).

The water content during the dehydration process was calculated using equation (1), with the initial fresh mass (saturated) used as a reference to derive the maximum water content:

(Eq. 1) $$\text{Water}\;\text{content}=\frac{\text{Fresh}\;\text{mass}-\text{Dry}\;\text{mass}}{\text{Dry}\;\text{mass}}$$

Exponential decay regressions were employed to fit the water loss curves of the samples as:

(Eq. 2) $$\text{Water}\;\text{content}=y+a*{e\;}^{\left(-\text{DC}*t\right)}$$

where t represents the measurement time, and a and y are coefficients. The parameter DC denotes the water loss decaying constant, with higher values indicating faster water loss rates (Song et al., 2015).

### 2.3 Measurements of morphological and anatomical traits

Another three medium-sized shoots from each sample were selected to measure morphological and anatomical traits. Data from these three shoots were averaged to represent each sample.

For each shoot, after full rehydration, the aboveground stem length and leaf number were recorded under a dissecting microscope (SZX7, Olympus, Japan). Leaf frequency was then derived by dividing the leaf number by the stem length. A single cell layered transverse section of the stem was cut using a razor blade at the mid-region and photographed under a light microscope (BX51, Olympus, Japan) at 400× magnification. The software ImageJ (National Institutes of Health, USA) was used to measure the circumference and area of stem transverse section, as well as the areas of the transport cells (the sum of the cortex and strands) and epidermis within the cross-section to calculate their area ratio (Fig. S2) (He et al., 2023). The shape index of the stem transverse section (with values increasing from 1 for a circle to higher values for irregular shapes) was calculated as follows:

(Eq. 3) $$\text{Shape}\;\text{index}=\frac{\text{Circumference}}{2*\sqrt{\pi *\text{Area}}}$$

Leaves from the upper one-third region of each shoot were stripped and medium-sized leaves were selected and mounted as temporary slides. Microscopic images were captured at 200× magnification for the entire leaf, and at 400× magnification to observe the cells from the leaf base, midrib, and tip regions. The areas and shape index of the leaf and individual leaf cells from different regions were measured using the software ImageJ. It should be noted that we only measured the leaf base cells of *Pogonatum* *inflexum*, as its middle and tip regions are composed of multiple layers of cells (Fig. S3B). In the center of each leaf region, we selected a 100 μm × 100 μm square, measured the number of cells within this area, and calculated the cell density per unit area (Fig. S3C). To calculate shape index, we measured the perimeters and areas of 5–10 medium-sized cells. To calculate the average intercellular space, we measured the total distance between 5 and 10 adjacent cells, subtracted the sum of the lengths of these cells, and calculated the average intercellular space (Fig. S3D).

Transverse sections were taken from the mid-region of the leaves. For *Pogonatum* *inflexum*, the average length of the lamellae was measured (Fig. S4). For *Hyophila* *propagulifera*, the area of the midrib’s transverse section was measured, the hydroid strands were identified, and the proportion of the midrib that these strands occupy was calculated (Fig. S5). The leaf thickness of these samples was measured (specifically the midrib thickness for *H*. *propagulifera*). A full list of the studied traits and their descriptions can be found in Table 1.

**Table 1:** Descriptions of the studied traits of *Hyophila propagulifera* and *Pogonatum inflexum*.

Category	Trait	Description	Unit
Water relations	Maximum water content	Water holding capacity	%
	Water loss decaying constant	Water retention capacity	─
Shoot & stem	Shoot mass	The current year’s shoots were selected for measurements	mg
	Stem length		mm
	Stem transverse area	Area of stem transverse section at the middle region	μm^2^
	Stem transverse shape index	$\text{Circumference}/\left(2*\sqrt{\pi *Area}\right)$	─
	Stem transverse transport cells/epidermis area	Area ratio of transport cells to epidermis in stem transverse section	─
	Leaf frequency	Number of leaves per stem length	Leaves mm^−1^
Leaf	Leaf area	─	mm^2^
	Leaf shape index	Calculated as stem transverse shape index above	─
	Leaf thickness	The thickness of transverse section of leaf	μm
	**Lamella length**^a^	Average length of lamellae at the middle range of the leaf	μm
	Leaf midrib transverse area	Transverse area of leaf midrib	μm^2^
	Proportion of hydroid strand in midrib	Area proportion of hydroid strands in leaf midrib transverse section	%
Leaf cell	Leaf base cell area	Average cell area in leaf base	μm^2^
	Leaf base cell shape index	Average cell shape index in leaf base	─
	Leaf base cell intercellular space	Average cell intercellular in leaf base	μm
	Leaf base cell density	Cell density in leaf base	Cells mm^−2^
	Leaf middle cell area	Average cell area in leaf middle region	μm^2^
	Leaf middle cell shape index	Average cell shape index in leaf middle region	─
	Leaf middle cell intercellular space	Average cell intercellular in leaf middle region	μm
	Leaf middle cell density	Cell density in leaf middle region	Cells mm^−2^
	Leaf tip cell area	Average cell area in leaf tip	μm^2^
	Leaf tip cell shape index	Average cell shape index in leaf tip	─
	Leaf tip cell intercellular space	Average cell intercellular in leaf tip	μm
	Leaf tip cell density	Cell density in leaf tip	Cells mm^−2^

a Lamella length (in bold) was measured only for *Pogonatum inflexum*, while the underlined traits were measured only for *Hyophila propagulifera*.

### 2.4 Data analysis

The coefficient of variation for each trait was first calculated. Prior to the subsequent analyses, we assessed the distribution of all traits. Those traits that violated the normality assumption (e.g., leaf cell area and cell density, which were predominantly right-skewed) were log_10_-transformed. We then used Linear Mixed Models (LMMs) to evaluate the effects of individual predictors (including both latitude and environmental factors) on individual traits, with ‘island’ included as a random factor. The validity of the final models was confirmed through checks for residual normality and homoscedasticity, employing Shapiro–Wilk tests (*p* > 0.05) alongside visual inspection of normal Q–Q and residual-versus-fitted plots.

To address potential multicollinearities among latitude and environmental factors, we performed a Spearman correlation analysis. It revealed that with decreasing latitude, MAT, MAP, and UVB increase, whereas MDR decreases (Fig. S6). Therefore, Boosted Regression Trees (BRT), which employ recursive partitioning and are robust to multicollinearity, were used to evaluate the relative importance of each environmental factor (Elith et al., 2008). All environmental factors were standardized to Z-scores to eliminate the influence of measurement units. The models were implemented using the *gbm.step* function in the R package ‘*dismo*’ with a Gaussian response distribution and the following parameters were set: learning rate = 0.001, bag fraction = 0.5, and tree complexity = 5. We fitted LMMs with the following specification: Trait ∼ MAT + MDR + MAP + UVB + (1|Island) to assess the combined effects of these environmental factors on specific trait. The coefficient of determination (marginal R^2^) was calculated using the ‘*glmm.hp*’ package (Lai et al., 2022, 2023).

## 3 Results

**Fig. 2:**
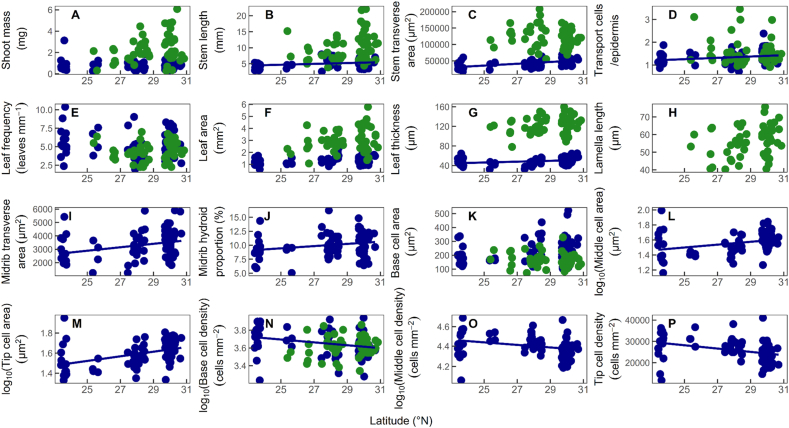
Relationships between latitude and functional traits of *Hyophila propagulifera* (blue) and *Pogonatum inflexum* (green). Solid lines indicate significant relationships identified by Linear Mixed Models. Detailed information is shown in Table A.3.

The ITV in studied traits ranged from 1.63 to 54.0% for *Pogonatum* *inflexum* and from 0.64 to 51.9% for *Hyophila* *propagulifera* (Fig. S7 and Table S2). For both species, the largest variation was recorded for shoot mass, whereas minimal variation was observed in the shape indices of the stem transverse section, leaf, and leaf cells from different regions (lower than 7.28%). Shoot mass and water relations (maximum water content and water decaying constant) exhibited no significant relationships with latitude or the studied environmental factors for either *H*. *propagulifera* or *P*. *inflexum* (Figs. 2a and S8; Table S3). In *H*. *propagulifera*, latitude was positively correlated with size-related traits, including stem length, stem transverse area, leaf thickness, and the areas of cells from the leaf middle and tip regions. Latitude was also correlated with traits reflecting transport capacity, including area ratio of transport cells to epidermis in stem transverse section, leaf midrib transverse area, and the proportion of hydroid strands in the midrib, but was negatively correlated with leaf cell densities (Fig. 2 and Table S3). No clear variations in traits were observed along latitude for *P*. *inflexum*.

**Fig. 3:**
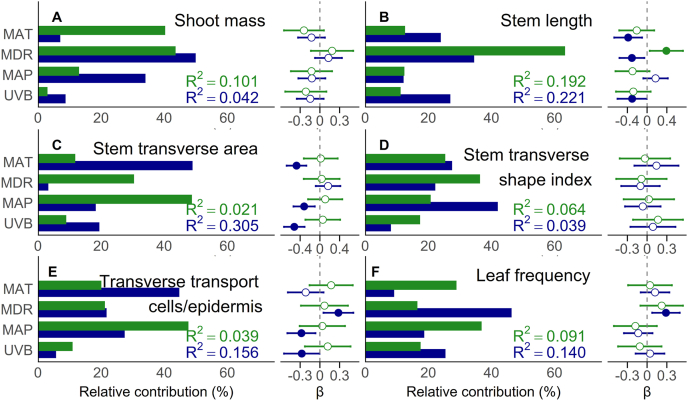
Relative contributions of environmental factors (mean annual temperature, MAT; mean annual precipitation, MAP; mean diurnal range, MDR; and ultraviolet B radiation, UVB) to shoot mass and stem traits of *Hyophila propagulifera* (blue) and *Pogonatum inflexum* (green). The total marginal coefficient of determination (R^2^) was calculated using hierarchical partitioning methods. Additionally, the standardized regression coefficients (β, with 95% confidence intervals) of the individual effects of each specific factor on each trait are shown. Solid points indicate significant relationships (*p* < 0.05). Detailed information is shown in Table S3.

**Fig. 4:**
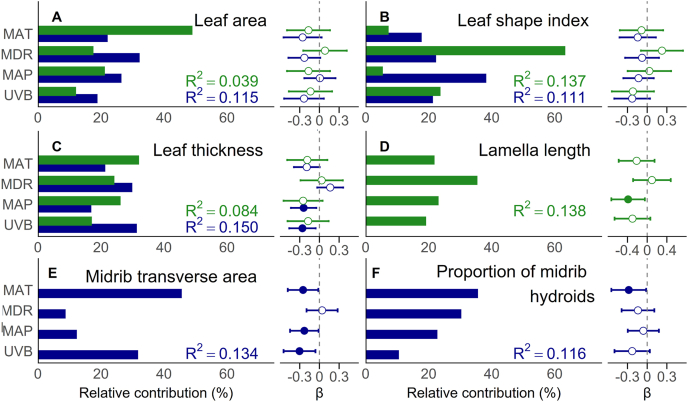
Relative contributions of environmental factors (mean annual temperature, MAT; mean annual precipitation, MAP; mean diurnal range, MDR; and ultraviolet B radiation, UVB) to leaf morphological and anatomical traits of *Hyophila propagulifera* (blue) and *Pogonatum inflexum* (green). The total marginal coefficient of determination (R^2^) was calculated using hierarchical partitioning methods. Additionally, the standardized regression coefficients (β, with 95% confidence intervals) of the individual effects of each specific factor on each trait are shown. Solid points indicate significant relationships (*p* < 0.05). Detailed information is shown in Table S3.

**Fig. 5:**
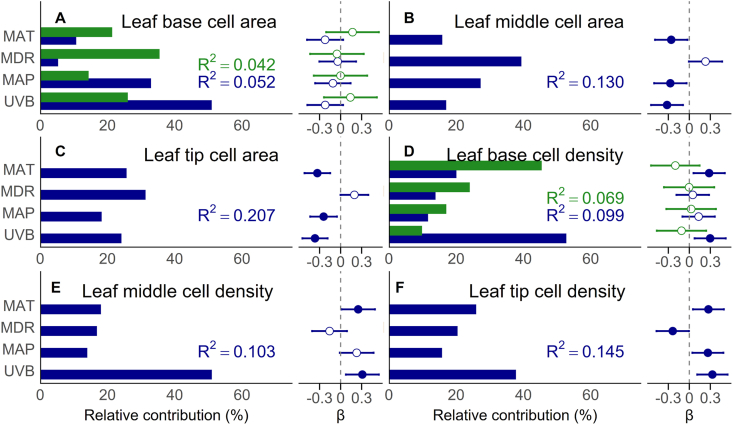
Relative contributions of environmental factors (mean annual temperature, MAT; mean annual precipitation, MAP; mean diurnal range, MDR; and ultraviolet B radiation, UVB) to the area and density of leaf cells at different regions (base, middle, tip) in *Hyophila propagulifera* (blue) and *Pogonatum inflexum* (green). The total marginal coefficient of determination (R^2^) was calculated using hierarchical partitioning methods. Additionally, the standardized regression coefficients (β, with 95% confidence intervals) of the individual effects of each specific factor on each trait are shown. Solid points indicate significant relationships (*p* < 0.05). Detailed information is shown in Table S3.

Morphological and anatomical variation within these two species was partially explained by MAT, MDR, MAP, and UVB, which together accounted for 2–31% of the observed ITV (Fig. 3, Fig. 4, Fig. 5). Given that the environmental factors in the study locations are significantly correlated with latitude, the relationships between these environmental factors and the studied traits are generally consistent with the latitudinal patterns of the traits described above. For *Hyophila* *propagulifera*, MAT, MAP, and UVB, which are negatively correlated with latitude, are generally negatively related to stem length, stem transverse area, leaf thickness, areas of leaf cells from middle and tip regions, area ratio of transport cells to epidermis, midrib transverse area, and proportion of hydroid strand, whereas they are mostly positively associated with cell densities. However, MDR, which is negatively correlated with latitude, is positively related to the area ratio of transport cells to epidermis and leaf frequency but negatively related to stem length and leaf tip cell density. For *Pogonatum* *inflexum*, most of the traits showed no significant relationships with these factors, except that stem length was positively related to MDR and lamella length was negatively related to MAP.

## 4 Discussion

### 4.1 Differential intraspecific variation among traits

For both species, the greatest variation of a functional trait was observed in shoot mass. This finding is consistent with previous analyses of terrestrial vascular plants, which showed that, due to more complex and varied acclimation processes across multiple organs, intraspecific variations in whole-plant traits are greater than those in leaf morphological traits (Siefert et al., 2015). In contrast, the lowest variation in functional traits were observed in the shape indices of stem transverse section, leaf, and leaf cells. This finding agrees with previous studies reporting that the coefficient of variation for the length-width ratio is relatively low compared to other morphological and anatomical traits in *Bryum argenteum* and *Didymodon rigidulus* (Wang et al., 2019a; Liu et al., 2021). Unlike the highly variable leaf shapes observed within vascular plant species (Nicotra et al., 2011), moss maintains a relatively stable shape despite environmental changes and size variations. Given its simple structures (e.g., non-lignified stems and single-cell-layer-thick leaves), a stable shape may be essential for moss to maintain basic structural integrity and physiological function under biophysical and geometric constraints (Siefert et al., 2015; Glime, 2017; Niklas and Cobb, 2017). The results of the quantitative analyses of leaf and cell shapes support the adoption of these traits as important taxonomic characteristics for mosses, although they are mostly described in qualitative terms in floras (Empain, 1987).

### 4.2 Trait variations along latitudinal and environmental gradients

The lack of significant associations between water relations and latitude/environmental factors for both species can be attributed to the fact that the southeastern coastal region of China has a subtropical monsoon climate, which is generally not water-limited. For instance, the MAP across the studied coastal islands ranges from 980 to 1600 mm. As a result, adjustments in water holding and retention capacities are less critical for the current mosses. However, in water-deficient environments, variations in water relations across environments are more pronounced, as Wan et al. (2025) found increasing water-holding capacity across successional stages for arid mosses in a temperate desert. Although no significant trend was observed in water relations, some anatomical traits that reflect the capacity of plants to transport or retain water showed clear variations along latitudinal and precipitation gradients. In *Hyophila* *propagulifera*, the water transport capacity, as indicated by stem cross-sectional area, ratio of stem transverse transport cells to epidermis, leaf midrib cross-sectional area, and proportion of hydroid strands in the midrib, decreased with decreasing latitude. The lower investment in endohydric transport structures, coupled with higher precipitation and shorter stem lengths, is probably because higher water availability and shorter transport distances facilitate a more prominent role for external capillary water conduction in fulfilling the mosses’ water requirements. For *Pogonatum* *inflexum*, the lamellae were longer with decreasing precipitation, which reduces water loss, increases the surface area, and reduces CO_2_ diffusion constraints, thereby promoting photosynthesis (Proctor, 2005; Proctor et al., 2007). Due to their small size, present studies on the water relations of bryophytes are still limited compared to vascular plants. Future studies should attempt to develop innovative, miniaturized instruments and novel experimental methods to enhance our understanding of the water use and adaptive mechanisms in bryophytes (Brodribb et al., 2020; Perera-Castro and Flexas, 2022).

With decreasing latitude, the stems of *Hyophila* *propagulifera* became shorter and thinner, likely because stronger UV-B radiation restricted stem growth and higher temperatures increased dark respiration rates that impacted the carbon balance (Fig. 2, Fig. 3) (Lappalainen et al., 2010; Wagner et al., 2013). It should be noted that such patterns are not static but highly contingent upon the specific study site. For example, in Qinghai-Xizang Plateau and Arctic regions, where low temperature is the predominant limiting factor, mosses growing in warmer sites possessed longer stems (Manolaki et al., 2022; Wang et al., 2024). Moreover, variation in leaf area was not correlated with either latitudes or environmental factors studied here, which also differs from previous studies that have shown leaf area to be negatively correlated with MAT and positively correlated with water availability (Printarakul and Jampeetong, 2020; Liu et al., 2021; Zhuo et al., 2023; Song et al., 2024b; Wang et al., 2024). Although the cell area of both the leaf middle and tip regions increased with increasing latitude, the densities of cells from different leaf regions all decreased, resulting in the relatively stable leaf area (Fig. 2F and L–P). The coordinated patterns of decreasing individual cell size and increasing cell density toward the south are assumed to serve to reduce UV damage and enhance water retention capacity (Fan et al., 2020; Liu et al., 2021).

The majority of studied traits in the terricolous *Pogonatum* *inflexum* appeared to be less influenced by latitude than those in the saxicolous *Hyophila* *propagulifera*, possibly due to their distinct growing substrates: soil provides plants with a more stable microclimatic temperature and humidity, as well as higher nutrient availability than rock does. These soil properties may even be the dominant factors in shaping the functional traits of terricolous mosses (Wang et al., 2019a, 2022a; Yang et al., 2025). On the other hand, the moss family Polytrichaceae has several unique characteristics compared to other mosses, such as lamellae on the leaf, surface wax, complex and efficient transport structures, and relatively high photosynthetic capacities (Wang et al., 2017, 2022b; Brodribb et al., 2020; Song et al., 2024a). These structural and physiological divergences, along with their species-specific ITV patterns, may explain why *P*. *inflexum* is capable of forming pioneer vegetation in disturbed habitats, whereas *H*. *propagulifera* predominantly occupies moist microsites.

Although environmental factors were significantly correlated with variation in several functional traits (especially for *Hyophila* *propagulifera*), the explanatory power (marginal R^2^) of these environmental factors was lower than 31%. This is partly because the use of mixed models, which accounts for the complexity of random effects, results in a more conservative estimation of the contribution of fixed effects (Nakagawa and Schielzeth, 2013). In addition, this illustrates the complexity and uniqueness in studying biogeographical variations and factors that drive bryophyte functional traits: 1) local microclimates and soil properties can better reflect variations in plant functional traits than data extracted from global models, especially given the small stature of bryophytes (Wang et al., 2026). However, obtaining a complete dataset of the heterogeneous environmental conditions across all sites is highly labor-intensive and time-consuming for large-scale biogeographical investigations; 2) the physiological activities of poikilohydric plants are highly sensitive to variations in environmental conditions. Therefore, climatic events, such as frequent wetting and drying cycles, may have a more pronounced effect on the growth and development of bryophytes than long-term averages, such as MAT and MAP (Stark et al., 2011; Nikolić et al., 2024).

## 5 Conclusion

Here we found that the highest intraspecific variation observed in two moss species collected from the coastal islands was for shoot mass, whereas the shapes of the stem transverse section, leaf, and leaf cells were relatively conserved. With increasing latitude, the stem size and internal transport capacity of *Hyophila* *propagulifera* increase, and the lamellae of *Pogonatum* *inflexum* become longer. However, the water holding and retention capacities were found to be unrelated to latitudinal and environmental gradients for both species.

We recommend that future bryophyte functional adaptation studies carefully consider the choice of specific environmental factors and their explanatory power for traits, which has been a much-discussed issue across scales (Collart et al., 2024). For the ecophysiology of poikilohydries, e.g., Alatalo et al. (2014) suggested that climatic events might be the primary determinants, whereas Stanton et al. (2023) indicated that vapor pressure deficits and air humidity play crucial roles. Based on the biogeographical investigations, more long-term *in situ* monitoring or controlled experiments are needed to exclude heterogeneous external disturbances, focus on the individual effect of a specific environmental factor, and thereby identify the key influences for mosses in specific habitats and uncover the underlying mechanisms (Bolnick et al., 2011; Nikolić et al., 2024). In addition, incorporating bryophytes into current terrestrial ecosystem models remains a significant challenge. To address this, it is essential to clarify the associations between bryophyte response traits, such as morphological and anatomical traits that indicate plant adaptations to the environment, and effect traits, such as water relations that reflect the ecosystem service functioning of water interception (Violle et al., 2007; Stanton and Coe, 2021). Moreover, more attention should be paid to bryophyte community structure metrics, such as biomass, cover, and community-weighted mean, in order to accurately quantify their ecological contributions and improve existing terrestrial ecosystem biogeochemical models (Wang et al., 2025b).

## Data availability statement

The data that support the findings of this study are openly available in the Science Data Bank at https://doi.org/10.57760/sciencedb.34409.

## CRediT authorship contribution statement

Zhe Wang: Conceptualization, Formal analysis, Methodology, Visualization, Writing - original draft, Writing - review & editing. Sheng-Xuan Cai: Investigation, Validation, Writing - original draft. Jing-Rou Yu: Investigation, Validation. Dan–Dan Li: Investigation, Methodology, Writing - review & editing. Xue-Ping Lai: Investigation. Ling-Ao Yang: Investigation. Shui-Liang Guo: Investigation, Writing - review & editing. Jing Yu: Conceptualization, Funding acquisition, Investigation, Supervision, Writing - review & editing.

## Declaration of competing interest

The authors declare that they have no known competing financial interests or personal relationships that could have appeared to influence the work reported in this paper.
