# Supplementary material for: Distinct intraspecific trait variations in two moss species: Insights from a latitudinal investigation across 66 coastal islands
Source: Plant Divers. 2026 Jan 2;48(3):567–75. doi: 10.1016/j.pld.2025.12.016 (PMC13250291; doi:10.1016/j.pld.2025.12.016)

## Supplementary data

Title: Distinct intraspecific trait variations in two moss species: Insights from a latitudinal investigation across 66 coastal islands

Authors: Zhe Wang, Sheng-Xuan Cai, Jing-Rou Yu, Dan-Dan Li, Xue-Ping Lai, Ling-Ao Yang, Shui-Liang Guo, Jing Yu

### Table S1

Geographical coordinates and environmental conditions of the sampling islands.

| Variable | Mean±SD | Median | Range | CV (%) |
| --- | --- | --- | --- | --- |
| Longitude (°E) | ─ | 122.03 | 116.96―122.78 | ─ |
| Latitude (°N) | ─ | 29.7 | 23.42―30.85 | ─ |
| Elevation (m) | 39.88±38.71 | 28.67 | 1―179 | ─ |
| Mean annual temperature (°C) | 17.34±1.48 | 16.78 | 15.21―21.59 | 8.53 |
| Mean diurnal range (°C) | 5.48±0.77 | 5.5 | 3.88―6.74 | 14.01 |
| Mean annual precipitation (mm) | 1323.87±153.28 | 1273 | 981―1595 | 11.58 |
| Ultraviolet B radiation (J m^-2^ day^-1^) | 3451.98±247.29 | 3355.48 | 3235.11―4109.8 | 7.16 |

### Table S2

Trait variations of *Pogonatum inflexum* (*n*=56) and *Hyophila propagulifera* (*n*=79) collected from the southeast coastal islands of China.

|  |  | *Pogonatum inflexum* | | *Hyophila propagulifera* | |
| --- | --- | --- | --- | --- | --- |
| Variable | Unit | Mean±SD(Median) | Range | Mean±SD(Median) | Range |
| Maximum water content | % | 320.84±94.77(302.5) | 153―644 | 242.2±102.02(231) | 101―822 |
| Water decaying constant | ─ | 0.05±0.02(0.05) | 0.03―0.13 | 0.19±0.06(0.2) | 0.03―0.34 |
| Shoot mass | mg | 2.42±1.31(2.12) | 0.28―6.08 | 0.83±0.43(0.74) | 0.21―3.13 |
| Stem length | mm | 10.15±4.22(9.02) | 3.85―22.1 | 5.11±1.23(5.1) | 2.57―7.96 |
| Stem transverse area | μm^2^ | 122329±30682(119806) | 55483―208144 | 44146±12404(44419) | 17354―71401 |
| Stem transverse shape index | ─ | 1.07±0.02(1.07) | 1.02―1.12 | 1.04±0.01(1.04) | 1.02―1.07 |
| Stem transverse transport cells / epidermis | ─ | 1.54±0.54(1.37) | 0.91―3.46 | 1.35±0.28(1.33) | 0.71―2.38 |
| Leaf frequency | leaves mm^-1^ | 4.46±1.23(4.38) | 2.25―7 | 5.27±1.72(5.24) | 1.93―10.4 |
| Leaf area | mm^2^ | 3.01±0.92(2.92) | 1.07―5.79 | 1.32±0.29(1.3) | 0.58―2.34 |
| Leaf shape index | ─ | 1.87±0.14(1.85) | 1.63―2.25 | 1.27±0.04(1.26) | 1.19―1.36 |
| Leaf thickness | μm | 122.69±16.52(122.59) | 78.01―159.94 | 48.89±6.83(48.61) | 32.15―64.88 |
| Lamella length | μm | 56.08±8.65(56.14) | 40.29―75.74 | ― | ― |
| Midrib transverse area | μm^2^ | ― | ― | 3295±1036 (3169) | 1235―5903 |
| Proportion of hydroids | % | ― | ― | 10.06±2.14(9.78) | 5.05―16.22 |
| Leaf base cell area | μm^2^ | 164.71±48.28(166.4) | 72.53―328.86 | 222.56±82.9(204.58) | 102.73―522.76 |
| Leaf base cell shape index | ─ | 1.44±0.2(1.42) | 1.04―1.92 | 1.42±0.18(1.43) | 0.98―1.79 |
| Leaf base cell intercellular | μm | 1.8±0.34(1.79) | 0.9―2.61 | 2.35±0.49(2.32) | 1.6―4.46 |
| Leaf base cell density | cells mm^-2^ | 4324±1113(4178) | 2199―7256 | 4633±1449(4300) | 1720―8700 |
| Leaf middle cell area | μm^2^ | ― | ― | 38.26±13.38(36.44) | 14.48―98.34 |
| Leaf middle cell shape index | ─ | ― | ― | 1.04±0.01(1.04) | 1.03―1.06 |
| Leaf middle cell intercellular | μm | ― | ― | 1.65±0.29(1.65) | 1.05―2.38 |
| Leaf middle cell density | cells mm^-2^ | ― | ― | 25910±6738 (24567) | 11733―49500 |
| Leaf tip cell area | μm^2^ | ― | ― | 40.95±12.1(40.31) | 21.37―89.25 |
| Leaf tip cell shape index | ─ | ― | ― | 1.04±0.01(1.04) | 1.03―1.06 |
| Leaf tip cell intercellular | μm | ― | ― | 1.75±0.33(1.73) | 0.94―2.96 |
| Leaf tip cell density | cells mm^-2^ | ― | ― | 25773±5623(26033) | 11733―41067 |

### Table S3

Effects of latitude and environmental factors on the studied traits for the two moss species collected from the southeast coastal islands of China. Shown are the *p*-values calculated using Linear Mixed Models (LMM) and the slopes for significant effects (*p* ≤ 0.05).

| Factor | Trait | *Hyophila propagulifera* | | *Pogonatum inflexum* | |
| --- | --- | --- | --- | --- | --- |
|  |  | p-GLMM | GLMM slope | p-GLMM | GLMM slope |
| Latitude | Maximum water content | 0.758 | ― | 0.875 | ― |
| MAT |  | 0.814 | ― | 0.303 | ― |
| MDR |  | 0.830 | ― | 0.260 | ― |
| MAP |  | 0.775 | ― | 0.643 | ― |
| UVB |  | 0.884 | ― | 0.759 | ― |
| Latitude | Water loss decaying constant | 0.115 | ― | 0.834 | ― |
| MAT |  | 0.052 | ― | 0.306 | ― |
| MDR |  | 0.859 | ― | 0.504 | ― |
| MAP |  | 0.637 | ― | 0.514 | ― |
| UVB |  | 0.084 | ― | 0.629 | ― |
| Latitude | Shoot mass | 0.176 | ― | 0.097 | ― |
| MAT |  | 0.215 | ― | 0.096 | ― |
| MDR |  | 0.203 | ― | 0.197 | ― |
| MAP |  | 0.237 | ― | 0.345 | ― |
| UVB |  | 0.154 | ― | 0.140 | ― |
| Latitude | Stem length | **0.041** | 0.145 | 0.087 | ― |
| MAT |  | **0.009** | -0.261 | 0.189 | ― |
| MDR |  | **0.018** | -0.493 | **0.031** | 0.083 |
| MAP |  | 0.169 | ― | 0.079 | ― |
| UVB |  | **0.045** | -0.001 | 0.097 | ― |
| Latitude | Stem transverse area | **< 0.001** | 2799.260 | 0.424 | ― |
| MAT |  | **< 0.001** | -3501.060 | 0.900 | ― |
| MDR |  | 0.185 | ― | 0.807 | ― |
| MAP |  | **0.005** | -29.840 | 0.457 | ― |
| UVB |  | **< 0.001** | -22.790 | 0.681 | ― |
| Latitude | Stem transverse shape index | 0.603 | ― | 0.432 | ― |
| MAT |  | 0.395 | ― | 0.837 | ― |
| MDR |  | 0.485 | ― | 0.589 | ― |
| MAP |  | 0.631 | ― | 0.863 | ― |
| UVB |  | 0.601 | ― | 0.312 | ― |
| Latitude | Stem transverse transport cells / epidermis area | **0.033** | 0.033 | 0.260 | ― |
| MAT |  | 0.116 | ― | 0.237 | ― |
| MDR |  | **0.016** | 0.108 | 0.637 | ― |
| MAP |  | **0.012** | -0.001 | 0.791 | ― |
| UVB |  | **0.046** | -0.0003 | 0.416 | ― |
| Latitude | Leaf frequency | 0.549 | ― | 0.380 | ― |
| MAT |  | 0.268 | ― | 0.748 | ― |
| MDR |  | **0.009** | 0.696 | 0.138 | ― |
| MAP |  | 0.217 | ― | 0.220 | ― |
| UVB |  | 0.684 | ― | 0.428 | ― |
| Latitude | Leaf area | 0.103 | ― | 0.164 | ― |
| MAT |  | 0.081 | ― | 0.249 | ― |
| MDR |  | 0.063 | ― | 0.539 | ― |
| MAP |  | 0.951 | ― | 0.249 | ― |
| UVB |  | 0.123 | ― | 0.336 | ― |
| Latitude | Leaf shape index | 0.119 | ― | 0.547 | ― |
| MAT |  | 0.300 | ― | 0.530 | ― |
| MDR |  | 0.546 | ― | 0.130 | ― |
| MAP |  | 0.258 | ― | 0.804 | ― |
| UVB |  | 0.097 | ― | 0.142 | ― |
| Latitude | Leaf thickness | **0.007** | 0.896 | 0.118 | ― |
| MAT |  | 0.055 | ― | 0.181 | ― |
| MDR |  | 0.101 | ― | 0.796 | ― |
| MAP |  | **0.016** | -0.014 | 0.084 | ― |
| UVB |  | **0.013** | -0.007 | 0.216 | ― |
| Latitude | Lamella length | ― | ― | 0.070 | ― |
| MAT |  | ― | ― | 0.192 | ― |
| MDR |  | ― | ― | 0.545 | ― |
| MAP |  | ― | ― | **0.033** | -0.019 |
| UVB |  | ― | ― | 0.088 | ― |
| Latitude | Leaf midrib transverse area | **0.008** | 146.865 | ― | ― |
| MAT |  | **0.037** | -155.814 | ― | ― |
| MDR |  | 0.731 | ― | ― | ― |
| MAP |  | **0.039** | -1.817 | ― | ― |
| UVB |  | **0.016** | -1.126 | ― | ― |
| Latitude | Proportion of hydroid strand | **0.042** | 0.237 | ― | ― |
| MAT |  | **0.039** | -0.342 | ― | ― |
| MDR |  | 0.262 | ― | ― | ― |
| MAP |  | 0.619 | ― | ― | ― |
| UVB |  | 0.094 | ― | ― | ― |
| Latitude | Leaf base cell area | 0.091 | ― | 0.367 | ― |
| MAT |  | 0.090 | ― | 0.298 | ― |
| MDR |  | 0.750 | ― | 0.737 | ― |
| MAP |  | 0.382 | ― | 0.991 | ― |
| UVB |  | 0.089 | ― | 0.385 | ― |
| Latitude | Leaf middle cell area | **0.006** | 0.019 | ― | ― |
| MAT |  | **0.030** | -0.021 | ― | ― |
| MDR |  | **0.056** | ― | ― | ― |
| MAP |  | **0.023** | -0.0003 | ― | ― |
| UVB |  | **0.008** | -0.0002 | ― | ― |
| Latitude | Leaf tip cell area | **< 0.001** | 0.023 | ― | ― |
| MAT |  | **0.001** | -0.028 | ― | ― |
| MDR |  | 0.052 | ― | ― | ― |
| MAP |  | **0.014** | -0.0003 | ― | ― |
| UVB |  | **< 0.001** | -0.0002 | ― | ― |
| Latitude | Leaf base cell intercellular | 0.162 | ― | 0.075 | ― |
| MAT |  | 0.388 | ― | 0.386 | ― |
| MDR |  | 0.213 | ― | 0.629 | ― |
| MAP |  | **0.034** | -0.0002 | 0.082 | ― |
| UVB |  | 0.161 | ― | 0.196 | ― |
| Latitude | Leaf middle cell intercellular | 0.242 | ― | ― | ― |
| MAT |  | 0.346 | ― | ― | ― |
| MDR |  | 0.453 | ― | ― | ― |
| MAP |  | 0.282 | ― | ― | ― |
| UVB |  | 0.179 | ― | ― | ― |
| Latitude | Leaf tip cell intercellular | 0.695 | ― | ― | ― |
| MAT |  | 0.724 | ― | ― | ― |
| MDR |  | 0.379 | ― | ― | ― |
| MAP |  | 0.072 | ― | ― | ― |
| UVB |  | 0.541 | ― | ― | ― |
| Latitude | Leaf base cell density | **0.013** | -0.016 | 0.402 | ― |
| MAT |  | **0.014** | 0.022 | 0.198 | ― |
| MDR |  | 0.674 | ― | 0.983 | ― |
| MAP |  | 0.251 | ― | 0.859 | ― |
| UVB |  | **0.010** | 0.0002 | 0.452 | ― |
| Latitude | Leaf middle cell density | **0.012** | -0.013 | ― | ― |
| MAT |  | **0.040** | 0.015 | ― | ― |
| MDR |  | 0.207 | ― | ― | ― |
| MAP |  | 0.062 | ― | ― | ― |
| UVB |  | **0.011** | 0.0001 | ― | ― |
| Latitude | Leaf tip cell density | **0.003** | -778.030 | ― | ― |
| MAT |  | **0.017** | 878.659 | ― | ― |
| MDR |  | **0.045** | -1800.990 | ― | ― |
| MAP |  | **0.020** | 10.798 | ― | ― |
| UVB |  | **0.004** | 6.308 | ― | ― |
| Latitude | Leaf base cell shape index | 0.064 | ― | 0.837 | ― |
| MAT |  | **0.034** | -0.031 | 0.690 | ― |
| MDR |  | 0.810 | ― | 0.767 | ― |
| MAP |  | 0.268 | ― | 0.796 | ― |
| UVB |  | 0.095 | ― | 0.785 | ― |
| Latitude | Leaf middle cell shape index | **0.002** | -0.001 | ― | ― |
| MAT |  | **0.018** | 0.001 | ― | ― |
| MDR |  | **0.048** | -0.002 | ― | ― |
| MAP |  | **0.001** | 0.00002 | ― | ― |
| UVB |  | **0.003** | 0.00001 | ― | ― |
| Latitude | Leaf tip cell shape index | 0.079 | ― | ― | ― |
| MAT |  | 0.361 | ― | ― | ― |
| MDR |  | **< 0.001** | -0.002 | ― | ― |
| MAP |  | **0.003** | 0.00001 | ― | ― |
| UVB |  | 0.118 | ― | ― | ― |

### Fig. S1

Detailed sampling sites of *Pogonatum inflexum* on Zhoushan (A) and Daishan (B) islands.


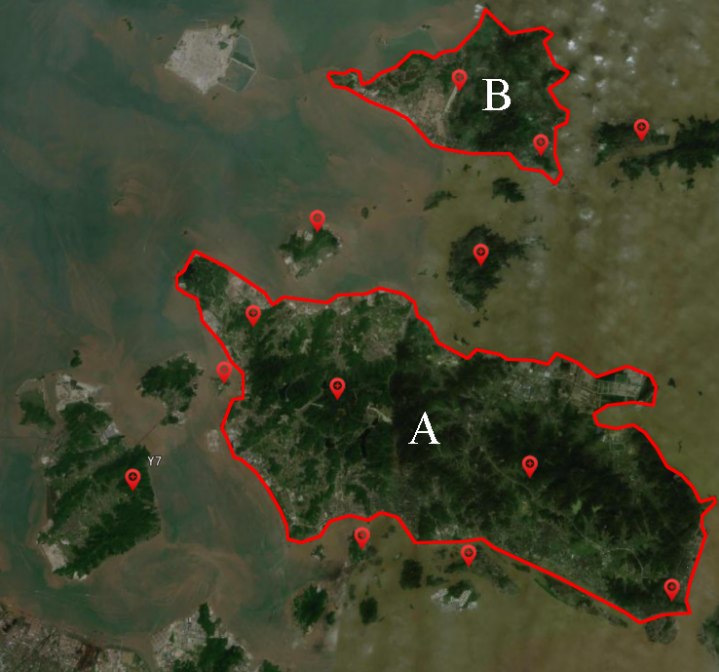


### *Fig. S2*

Stem transverse sections of *Hyophila propagulifera* (A) and *Pogonatum inflexum* (B). The area within the blue line indicates the transport cells, which include the cortex and strands, while the area between the red and blue lines represents the epidermis (B).

| A |
| --- |
| 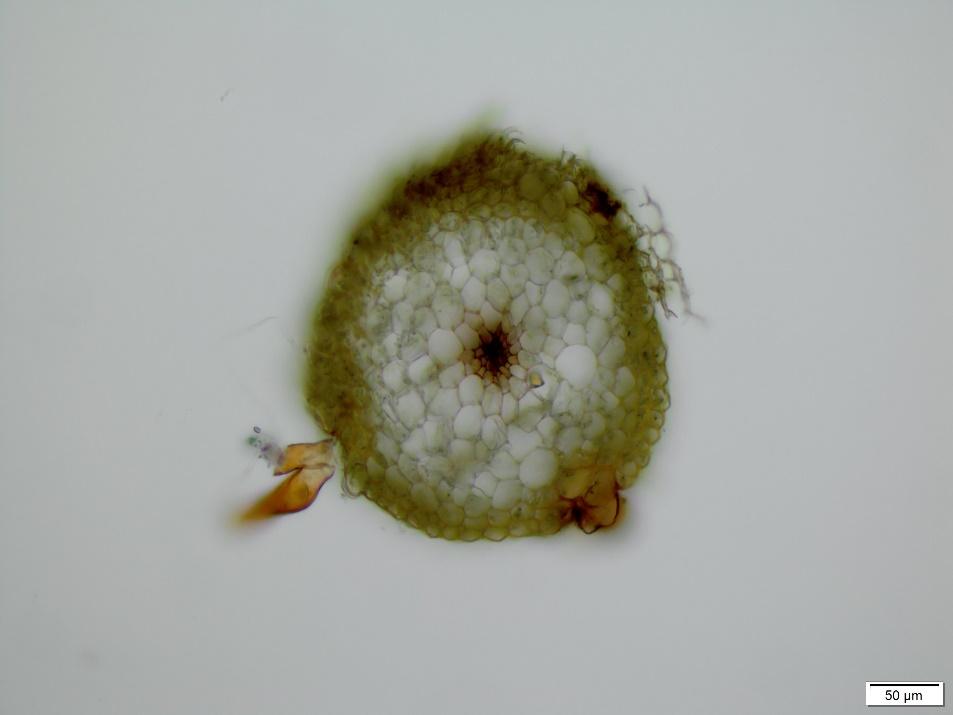 |
| B |
| 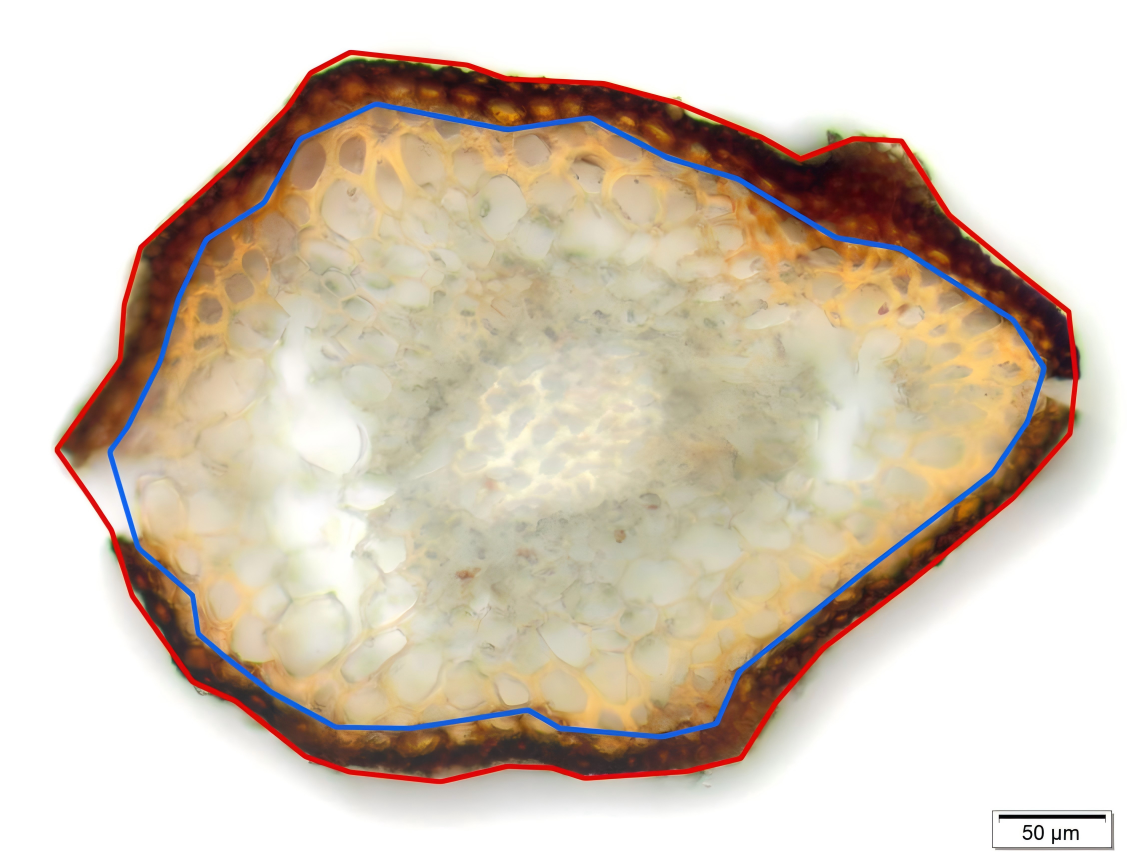 |

### Fig. S3

Leaves of *Hyophila propagulifera* (A) and *Pogonatum inflexum* (B), and the measurements of cell density (C) and cell intercellular space of *Hyophila propagulifera* (D).

| A | B |
| --- | --- |
| 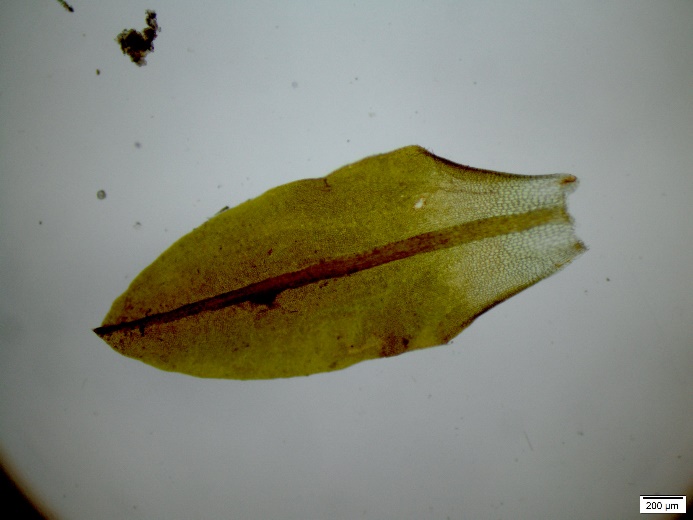 | 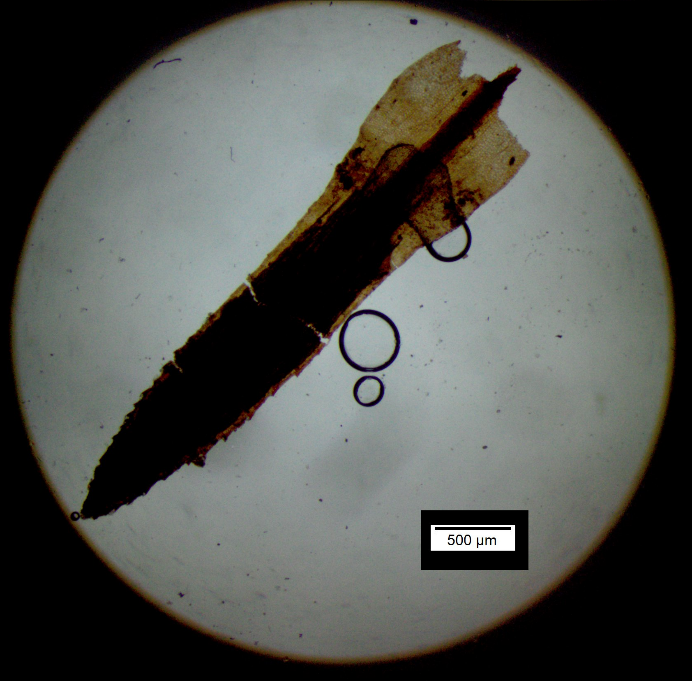 |
| C | D |
| 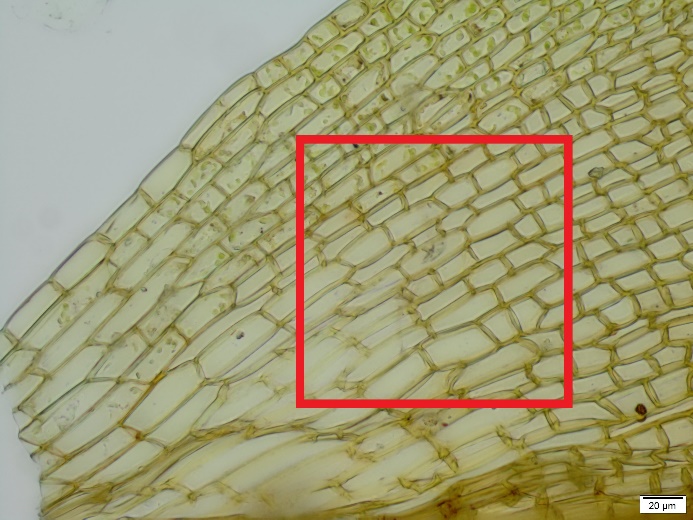 | 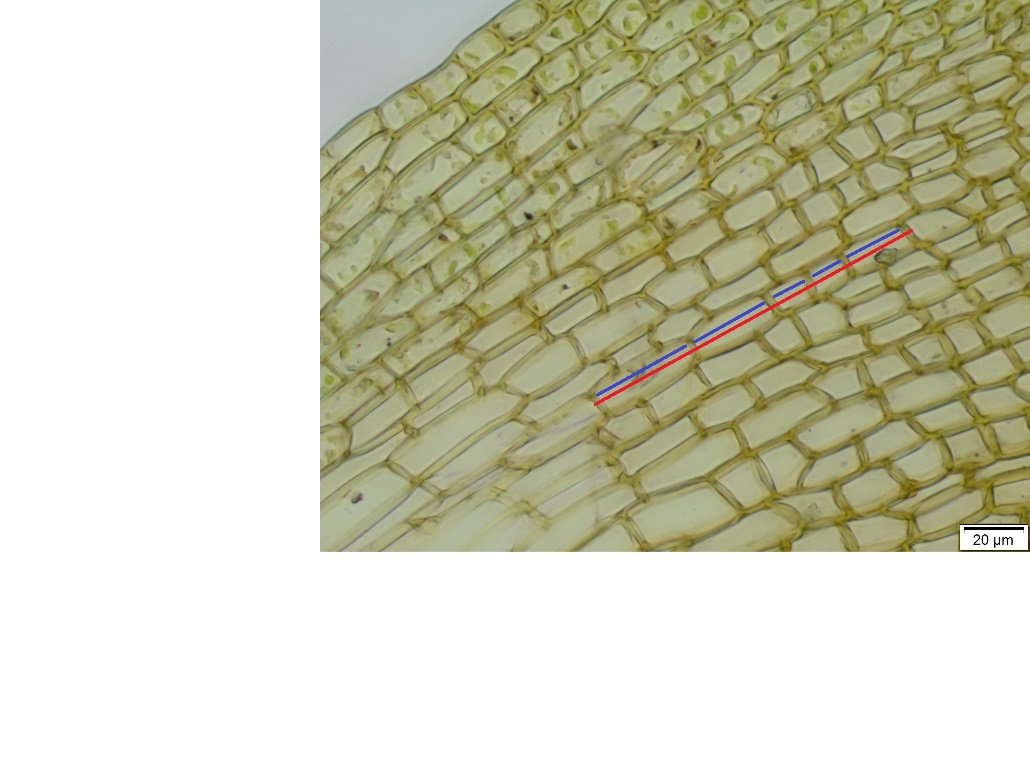 |

### Fig. S4

Leaf lamellae of *Pogonatum inflexum.*


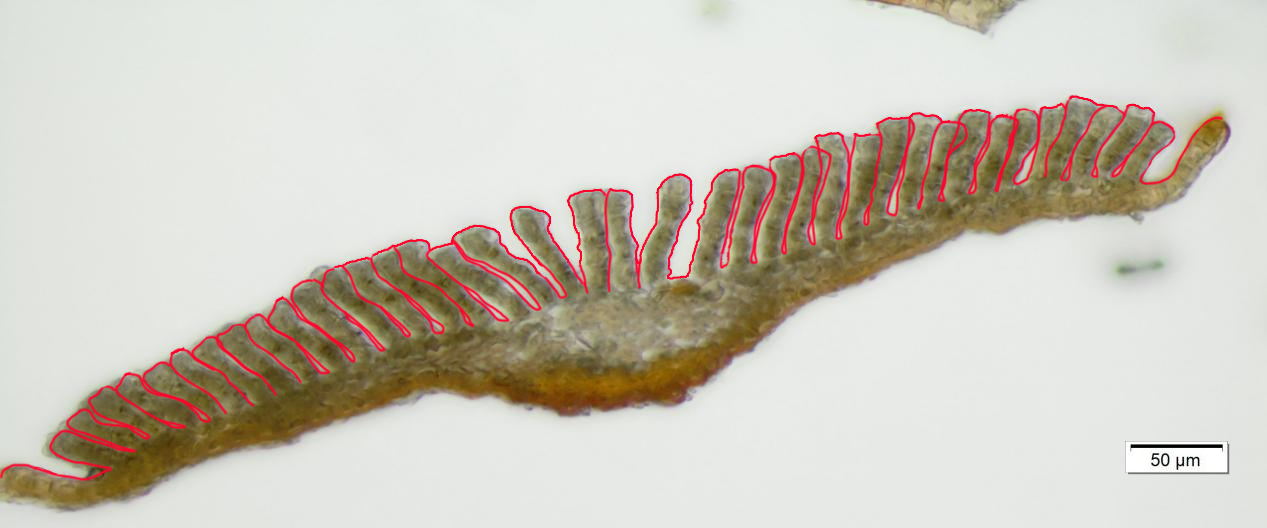


### *Fig. S5*

Measurements of the midrib transverse section of Hyophila propagulifera. The blue shapes represent the hydroids.


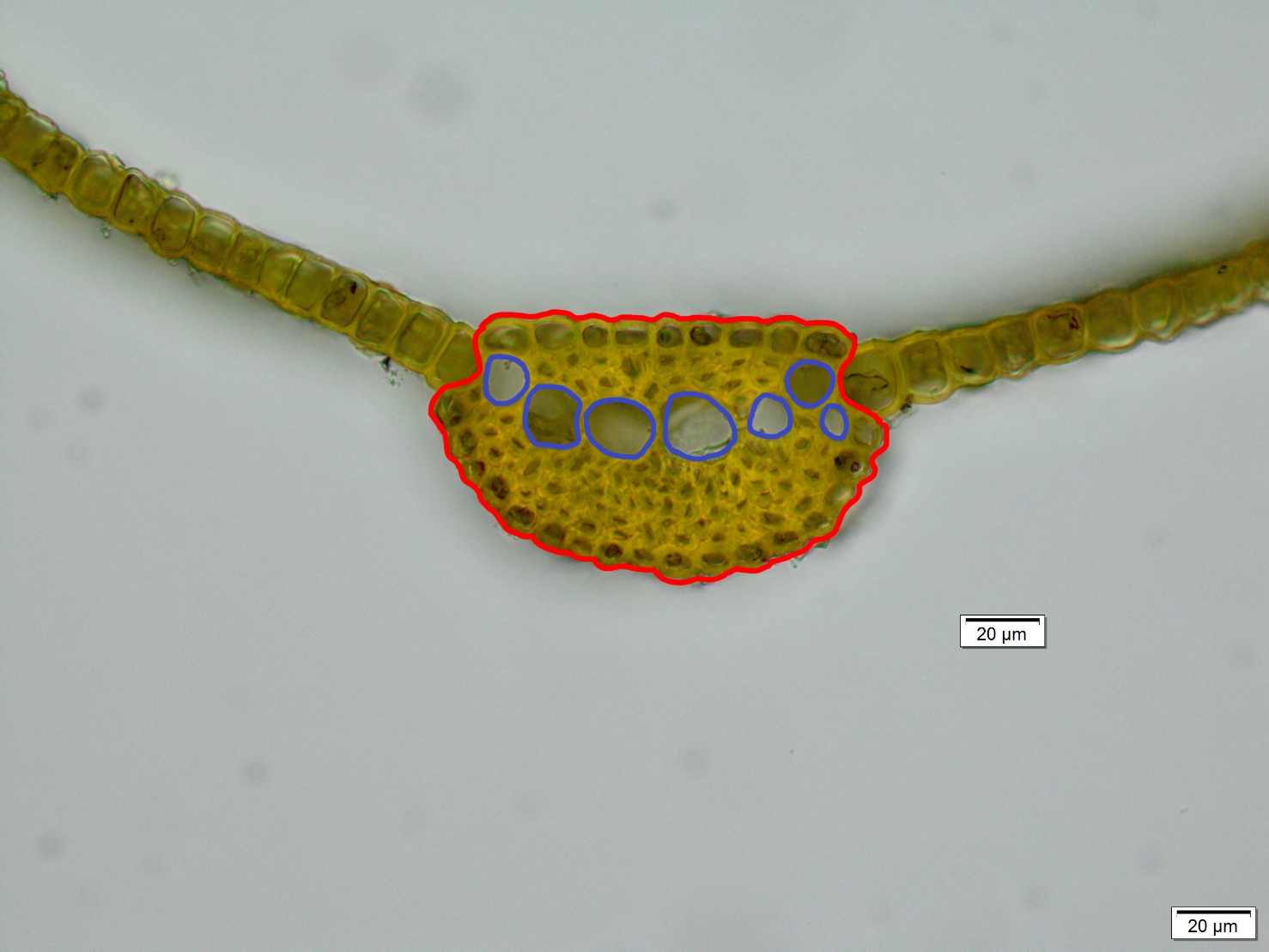


### Fig. S6

Spearman correlations among latitude (Lat.) and environmental factors (mean annual temperature (MAT) and precipitation (MAP), mean diurnal range (MDR), and ultraviolet B radiation (UVB)). The values below the variables (on the diagonal line) are Spearman correlation coefficients (*R*) and the “***” indicates significant bivariate correlation relationship (*p* ≤ 0.001).


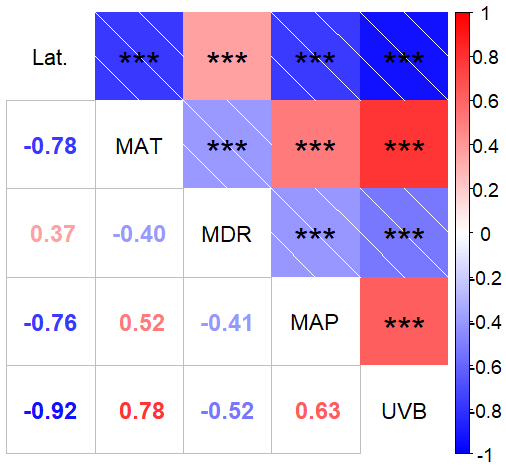


### Fig. S7

Coefficient of variation for the studied traits in *Pogonatum inflexum* and *Hyophila propagulifera*. Detailed information of the traits is provided in Tables 1 and S2.


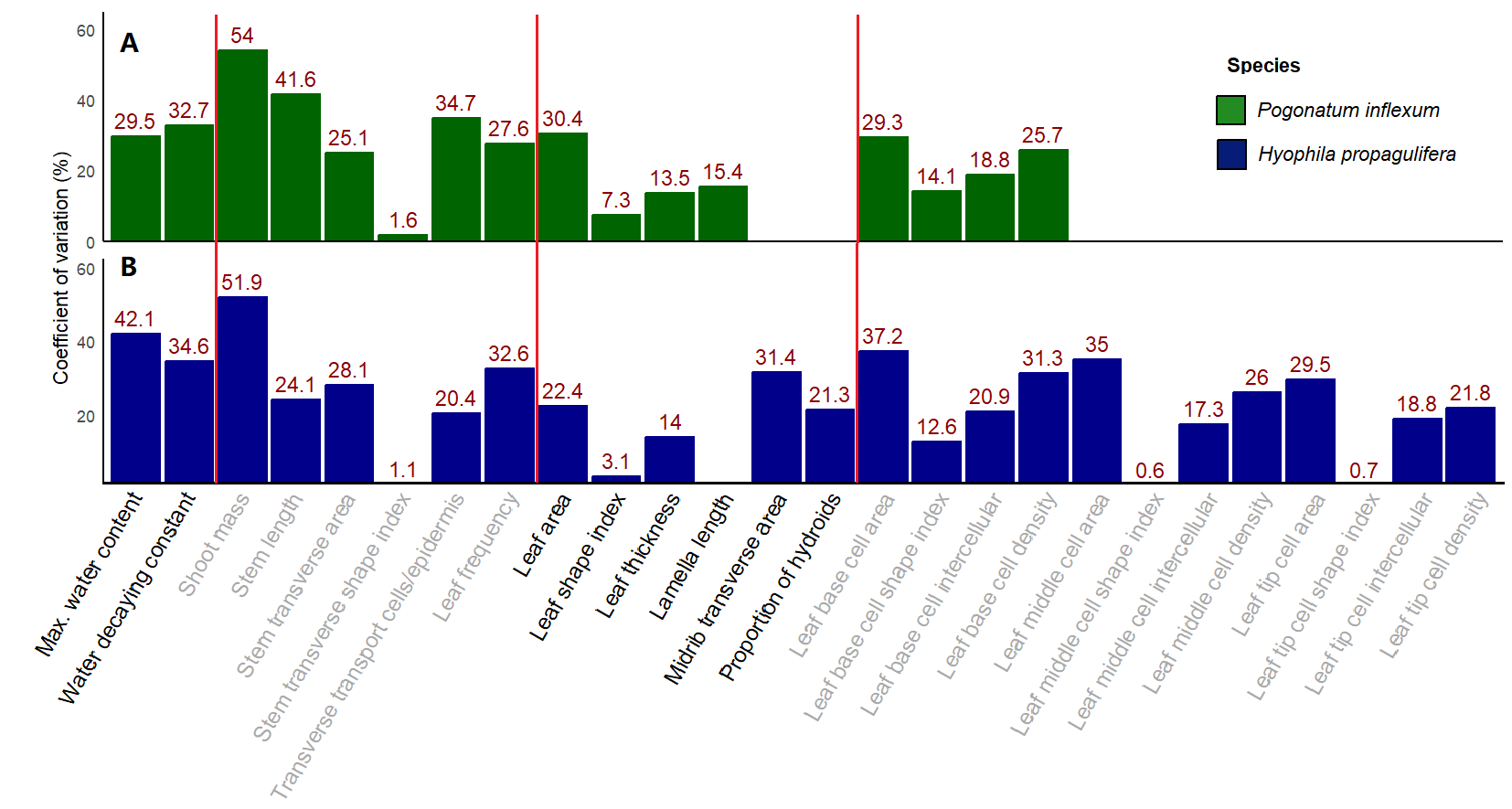


### Fig. S8

Relative contributions of environmental factors (mean annual temperature (MAT) and precipitation (MAP), mean diurnal range (MDR), and ultraviolet B radiation (UVB)) to the maximum water content and water decaying constant of *Hyophila propagulifera* (blue) and *Pogonatum inflexum* (green). The total marginal coefficient of determination (R²) was calculated using hierarchical partitioning methods. Additionally, the standardized regression coefficients (β, with 95% confidence intervals) of the individual effects of each specific factor on each trait are shown.
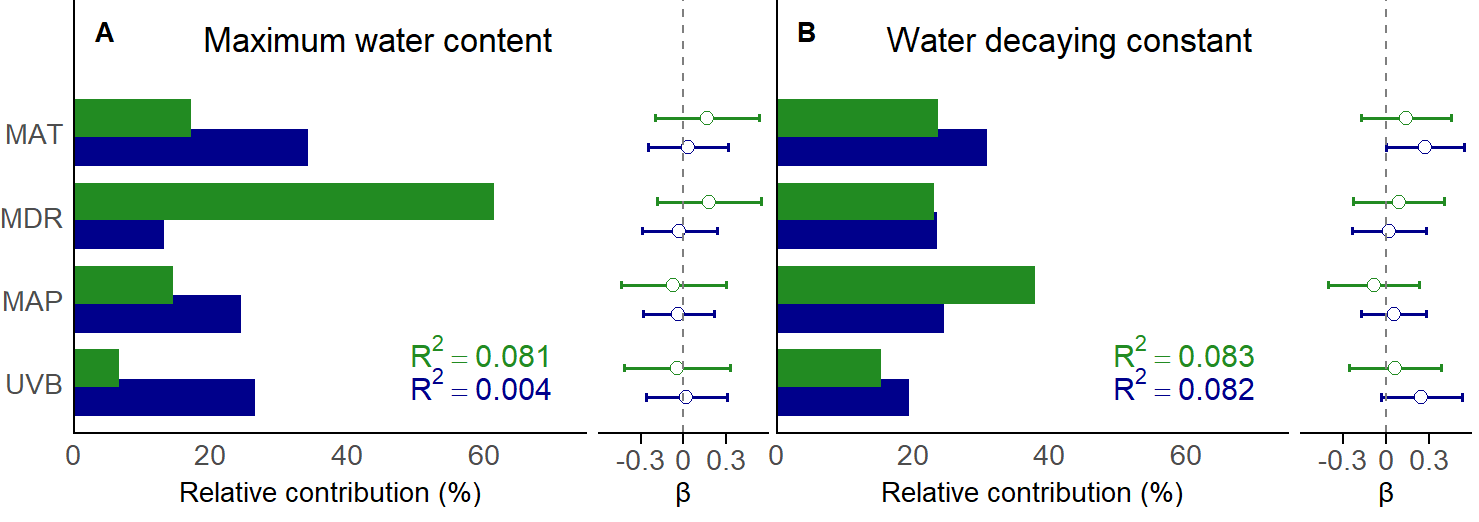

Supplement: Multimedia component 1 [file mmc1.docx]
